# Primary Antiphospholipid Syndrome Presenting With Life-Threatening Diffuse Alveolar Hemorrhage

**DOI:** 10.7759/cureus.93814

**Published:** 2025-10-04

**Authors:** João Casanova Pinto, Manuel G Costa, Beatriz Fernandes, Carlos Ramalheira

**Affiliations:** 1 Internal Medicine, Hospital de Cascais, Dr. José de Almeida, Lisbon, PRT; 2 Faculty of Medicine and Health Sciences, Universitat de Barcelona, Barcelona, ESP; 3 NOVA Medical School, Universidade Nova de Lisboa, Lisbon, PRT

**Keywords:** 2023 acr/eular classification criteria, antiphospholipid antibody, antiphospholipid antibody syndrome (aps), catastrophic antiphospholipid antibody syndrome, diffuse alveolar hemorrhage, primary antiphospholipid antibody syndrome, pulmonary capillaritis, venous thromboembolism

## Abstract

Antiphospholipid syndrome (APS)-associated diffuse alveolar hemorrhage (DAH) results from antiphospholipid-induced endothelial dysfunction, microvascular thrombosis, and complement activation. Management requires balancing thrombosis prevention and hemorrhage control. High-dose corticosteroids are the first-line therapy.

We report a case of a 33-year-old male presenting with progressive dyspnea, hemoptysis, and hypoxemia. Initial evaluation revealed thrombocytopenia, anemia, elevated inflammatory markers, and diffuse bilateral ground-glass opacities on a thoracic computed tomography, consistent with DAH. Autoimmune serology confirmed persistent positivity for lupus anticoagulant, anti-cardiolipin IgM, and anti-β2-glycoprotein-I IgM, fulfilling APS classification criteria. The patient developed respiratory failure, requiring mechanical ventilation and venovenous extracorporeal membrane oxygenation (VV-ECMO). High-dose corticosteroids were initiated, leading to gradual improvement. He was successfully extubated and discharged on long-term anticoagulation with warfarin. At four years of follow-up, he remains free of APS-related complications.

This case highlights the importance of recognizing APS as a cause of DAH, particularly in patients with unexplained pulmonary hemorrhage. Early diagnosis and aggressive therapy can improve outcomes.

## Introduction

Antiphospholipid syndrome (APS) is an autoimmune thrombo-inflammatory disorder characterized by recurrent thrombotic events and/or obstetric complications in individuals with persistently positive antiphospholipid antibodies [1,2]. It is frequently associated with systemic lupus erythematosus (SLE), affecting up to one-third of patients, but can also occur as a primary condition. A subset of APS patients (~1%) may develop catastrophic antiphospholipid syndrome (CAPS), a life-threatening variant marked by small-vessel thromboses with multi-organ failure [3]. APS prevalence is 40-50 cases per 100,000 people, with an annual incidence of one to two per 100,000, and it is more frequent in younger individuals [2].

Since 2006, diagnosis has relied on the revised Sapporo classification criteria, requiring clinical and laboratory findings [4]. While APS typically presents with vascular thrombosis, “non-criteria” manifestations are increasingly recognized, including diffuse alveolar hemorrhage (DAH), a rare but potentially fatal complication [5-7]. DAH, i.e., alveolar bleeding often causing acute respiratory failure, typically presents with cough, dyspnea, fever, and hemoptysis, and more commonly affects middle-aged men [8]. Laboratory abnormalities include hypoxemia and anemia, while imaging reveals diffuse, evolving alveolar infiltrates. As DAH is a diagnosis of exclusion, infection, pulmonary embolism, coagulopathy, and uremia must be ruled out. Bronchoalveolar lavage may detect hemosiderin-laden macrophages, while lung biopsy is reserved for selected cases. Alveolar hemorrhage and microvascular thrombosis, with or without pulmonary capillaritis, are the histopathologic hallmarks [8]. Although autoimmune diseases like SLE and vasculitis are common causes of DAH, APS-associated DAH remains an independent entity. It may be the initial presentation of APS [1,8]. The 2023 American College of Rheumatology/European Alliance of Associations for Rheumatology (ACR/EULAR) classification update has broadened APS recognition to include DAH as a possible manifestation [7]. While classification criteria do not confirm diagnosis, they aid in identifying complex APS cases where timely recognition is critical.

Notably, 46% of CAPS patients lack prior APS history [9], and 60% have no associated autoimmune disease [10]. Triggers include infection, surgery, malignancy, or ineffective anticoagulation, though 35% of cases occur without an identifiable trigger [10]. It commonly involves the kidneys (73%), lungs (60%), brain (56%), heart (50%), and skin (47%), with untreated mortality approaching 75% [10]. However, aggressive “triple therapy” (anticoagulation, high-dose corticosteroids, plasma exchange or intravenous immunoglobulin (IVIG) (± rituximab)) has significantly improved survival, reducing mortality to 30% [3]. Prompt high-dose intravenous corticosteroids are the cornerstone therapy for APS-associated DAH, particularly in cases of severe respiratory distress (e.g., methylprednisolone at 1 g/day for three to five days). Most patients respond well, although refractory cases may require rituximab or cyclophosphamide. Plasma exchange and IVIG have also demonstrated efficacy in corticosteroid-resistant cases [3]. A key challenge is anticoagulation: it may need temporary interruption during active bleeding and careful reintroduction once pulmonary stability is achieved. Furthermore, some patients relapse without sustained immunosuppressive therapy, highlighting the need for long-term management strategies like in other forms of pulmonary capillaritis [8].

Here, we present a case of primary APS with severe DAH, outlining diagnosis, pathophysiology, treatment, and outcomes, while emphasizing early, intensive management informed by recent evidence and guidelines.

This case was presented as an abstract/poster at the 20th European Congress of Internal Medicine in 2022.

## Case presentation

A 33-year-old male with a history of active smoking presented to the emergency department with a five-day history of dyspnea, cough, and hemoptysis. He also mentioned myalgia and malaise. He reported possible occupational exposure to rat feces and bleach. He denied significant medical history, recent medication use, or illicit drug consumption. On admission, the physical examination exhibited tachypnea (28 breaths per minute), hypoxemia (oxygen saturation of 90% while breathing room air), and mild tachycardia (102 beats per minute). Blood pressure was normal, and he was afebrile. Chest auscultation revealed bilateral coarse crackles and scattered rhonchi. Cardiovascular and abdominal examinations were unremarkable, and there were no cutaneous or joint abnormalities. Blood tests showed mild thrombocytopenia, neutrophil-predominant leukocytosis, and elevated inflammatory markers (Table 1). Despite initially being normal, hemoglobin dropped by > 3 g/dL (to 10.5 g/dL) in 12 hours. Thrombocytopenia also worsened (108 × 10⁹/L). The Coombs test was negative, and peripheral smear excluded microangiopathic hemolysis. Urinalysis showed mild proteinuria, without hematuria or dysmorphic erythrocytes.

**Table 1:** Blood test results on admission. ANA: antinuclear antibody; ANCA: antineutrophil cytoplasmic antibody; anti-CCP: antibody to cyclic citrullinated peptide; anti-dsDNA: antibody to double-stranded DNA; anti-GBM: antibody to glomerular basement membrane; anti-La/SSB: antibody to La/SSB antigen (Sjogren syndrome type B); anti-Ro/SSA: antibody to Ro/SSA antigen (Sjogren syndrome type A); IgG: immunoglobulin G; IgM: immunoglobulin M.

Blood test	Results (admission)	Normal range
C-reactive protein (mg/dL)	13.71	<0.5
Erythrocyte sedimentation rate (mm/h)	123	<15
D-dimer (ng/mL)	1360	<500
Fibrinogen (mg/dL)	645	200 – 400
Leucocytes (× 10⁹/L)	12,140	4,000 – 10,000
Hemoglobin (g/dL)	13.6	13.5 – 17.5
Platelet count (× 10⁹/L)	142	150 – 450
Albumin-to-creatinine ratio (mg/g creatinine)	202	<30
C3 (mg/dL)	131	90 – 180
C4 (mg/dL)	29	10 – 40
Lupus anticoagulant	Positive	Negative
Anti-cardiolipin IgM (MPL/mL)	30	<20
Anti-cardiolipin IgG (GPL/mL)	4.3	<20
Anti-β2-glycoprotein-I IgM (U/mL)	49	<20 or <99^th^ percentile
Anti-β2-glycoprotein-I IgG (U/mL)	3.3	<20 or <99^th^ percentile
Immunological study	Negative for ANA, anti-dsDNA, ANCA, anti-GBM, anti-Ro/SSA, anti-La/SSB, anti-CCP, anti-centromere, anti-RNA polymerase III, anti-topoisomerase I	

**Figure 1:**
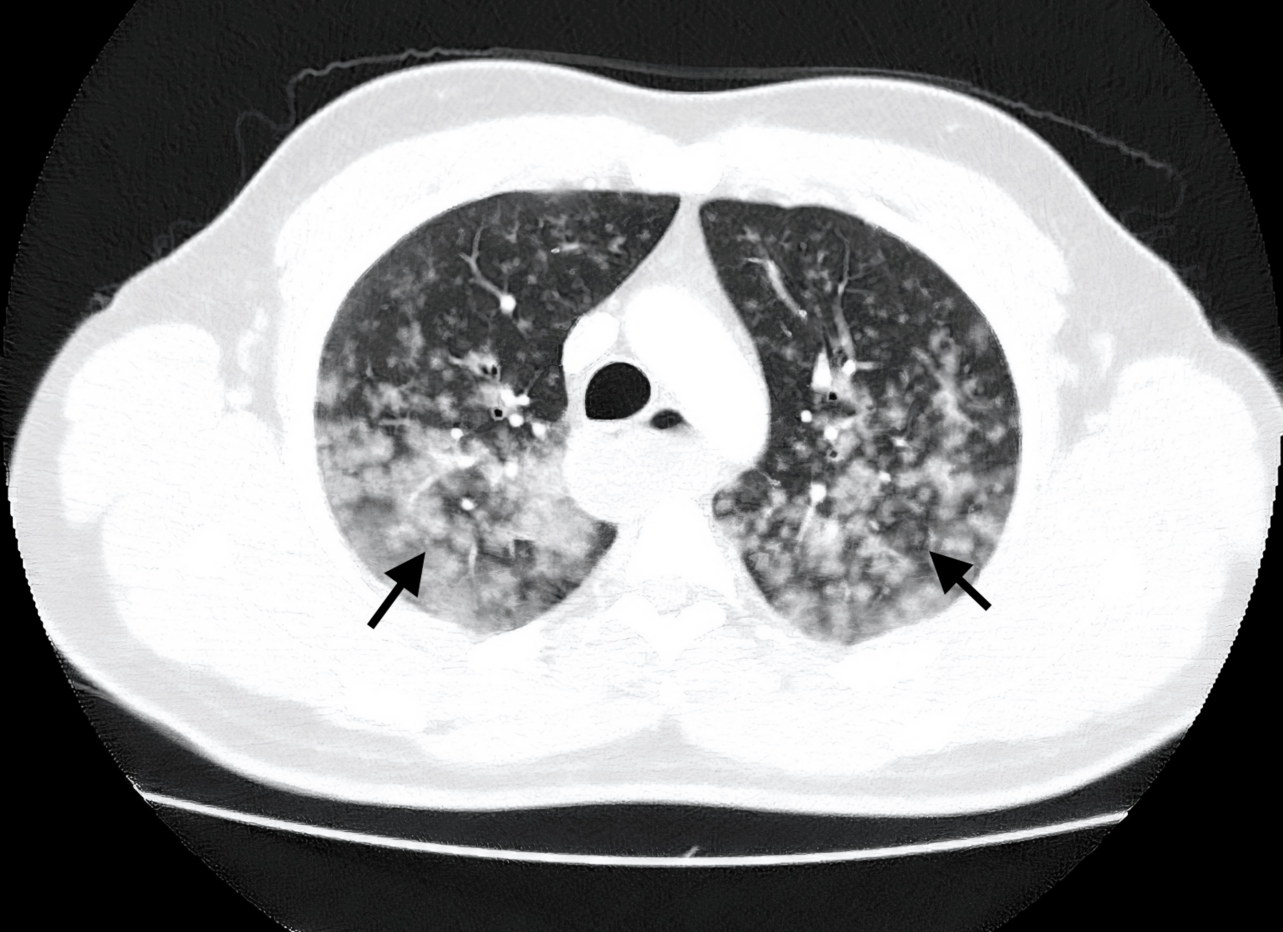
Axial non-enhanced CT image in lung window at the level of the aortic arch. There are bilateral ground glass opacities in a centrilobular and perivascular distribution in the posterior aspect of both lungs (arrows). These findings are compatible with diffuse pulmonary hemorrhage.

Thoracic CT angiography (CTA) revealed diffuse bilateral ground-glass opacities with a nodular pattern, consistent with DAH, while excluding pulmonary embolism and vascular malformations (Figure 1).

Autoimmune serology confirmed lupus anticoagulant, anti-cardiolipin IgM, and anti-β2-glycoprotein-I IgM positivity. All other autoantibodies were negative. Complement levels were normal. Within 12 hours, respiratory function rapidly deteriorated, requiring endotracheal intubation and intensive care unit (ICU) admission. Given a strong suspicion of immune-mediated DAH, pulse methylprednisolone (1 g/day for five days) was started. A whole-body CT scan ruled out malignancy, and bronchoscopy confirmed active alveolar hemorrhage with no endobronchial lesions. Extensive infectious workup was negative, including blood cultures, bronchoalveolar lavage (BAL), urinary antigen tests (*Streptococcus pneumoniae*, *Legionella pneumophila*), viral DNA panel (HIV, hepatitis C virus, SARS-CoV-2, influenza A/B), and serologies (*Mycoplasma pneumoniae*, *Chlamydia pneumoniae*, *Leptospira interrogans*). Toxicology screening for cocaine metabolites was also negative. Over the first 72 hours of hospitalization, hemoglobin declined to 7.8 g/dL, and transfusions were administered, but vasopressor support was not initiated. Despite high-dose corticosteroids, hypoxemia persisted despite conventional ventilation, leading to venovenous extracorporeal membrane oxygenation (VV-ECMO) initiation on day eight. Meanwhile, an incidental subacute thrombus was identified in the right internal jugular vein, with evidence of prior thrombosis in the left internal jugular vein, prompting therapeutic unfractionated heparin. ICU stay was complicated by a ventilator-associated pneumonia due to *Haemophilus influenzae*, and a *Klebsiella pneumoniae* bloodstream infection, treated with piperacillin-tazobactam for 14 days. Under VV-ECMO support, respiratory improvement allowed weaning and decannulation by day 11 and extubation on day 12. A second bronchoscopy revealed diffusely erythematous, friable mucosa with hematic BAL, but no ongoing hemorrhage. The patient underwent respiratory and motor rehabilitation. He was discharged on day 32, under warfarin to an international normalized ratio (INR) target of 2 to 3, and prednisone 20 mg/day, the latter tapered successfully over one month.

At 12 weeks, autoimmune testing confirmed persistently elevated anti-β2-glycoprotein-I IgM and anti-cardiolipin IgM, with negative lupus anticoagulant, fulfilling the revised Sydney APS classification criteria (Table 2) [4]. At four years of follow-up, the patient has remained free of APS-related complications under long-term warfarin therapy.

**Table 2:** Revised Sapporo classification criteria for antiphospholipid syndrome. * Definite antiphospholipid syndrome is present if at least one of the clinical criteria and one of the laboratory criteria are met. Source: Miyakis et al. [4].

Clinical criteria
Vascular thrombosis: ≥ 1 arterial, venous, or small vessel thrombosis
Pregnancy morbidity
- ≥ 1 fetal death (at or beyond the 10^th^ week of gestation)
- ≥ 1 premature birth before the 34^th^ week of gestation because of eclampsia, severe preeclampsia, or placental insufficiency
- ≥ 3 consecutive (pre) embryonic losses (before the 10^th^ week of gestation)
Laboratory criteria (on ≥ 2 occasions at least 12 weeks apart)*
- Lupus anticoagulant positivity
- Anticardiolipin antibody (IgG and/or IgM) in medium or high titer (i.e., > 40 or above the 99^th^ percentile)
- Anti-b2-glycoprotein-I antibody (IgG and/or IgM) in medium or high titer (i.e., above the 99^th^ percentile)

## Discussion

While DAH is more commonly associated with SLE and anti-neutrophil cytoplasmic antibodies (ANCA)-associated vasculitis, its occurrence in primary APS, even as an initial presentation, should not be overlooked [11,12]. Several mechanisms have been proposed to explain APS-associated DAH, including microvascular thrombosis, endothelial activation, and complement-driven capillaritis [13]. Antiphospholipid antibodies, particularly anti-β2GPI and anti-cardiolipin antibodies, induce endothelial dysfunction by upregulating tissue factor expression and promoting platelet activation, establishing a procoagulant state. In this patient, persistently elevated anti-β2GPI IgM likely contributed to pulmonary microthrombosis, ischemic endothelial injury, and secondary alveolar hemorrhage. Additionally, complement activation, especially via the C5a-C5aR axis, further increased endothelial permeability and promoted neutrophilic infiltration, culminating in capillaritis, a histopathological hallmark of immune-mediated DAH [13].

In this case, normal C3/C4 levels suggest localized pulmonary complement activation rather than systemic hypocomplementemia. Furthermore, the mild thrombocytopenia observed may be attributed to platelet consumption in microthrombi or secondary immune thrombocytopenia, which is supported by the absence of schistocytes and rapid steroid response. The clinical presentation of DAH, characterized by hemoptysis, dyspnea, hypoxemia, and diffuse alveolar opacities, mimics pneumonia, malignancies, pulmonary embolism, and other autoimmune diseases [11,12]. Excluding alternative causes through a comprehensive workup is therefore crucial. This patient's negative autoimmune markers for SLE and ANCA vasculitis, coupled with persistently positive antiphospholipid antibodies (aPL), strongly supported the diagnosis of APS-associated DAH. The 2023 ACR/EULAR classification criteria have broadened the recognized APS manifestations, allowing earlier identification of severe or atypical presentations, such as DAH [7]. This patient met both the Sydney APS criteria (persistent aPL, venous thrombosis) and the 2023 ACR/EULAR criteria (DAH, high-risk aPL profile). Although not diagnostic, these frameworks may help in deciding who may benefit from targeted investigations and early treatment.

Given the life-threatening nature of APS-associated DAH, its management requires balancing thrombosis prevention with hemorrhage control. High-dose corticosteroids remain the cornerstone, as they rapidly suppress aPL-mediated inflammation and bleeding. In this case, methylprednisolone (1 g/day for five days) was promptly initiated, consistent with the EULAR recommendations for severe APS [14]. For refractory cases, plasmapheresis, IVIG, rituximab, or cyclophosphamide may be considered [15]. Despite persistent hypoxemia, this patient responded to high-dose corticosteroids alone. However, had DAH been refractory, plasmapheresis or IVIG would have been justified. The nosocomial infections acquired during ICU admission highlight the susceptibility of critically ill APS patients to hospital-acquired infections and underscore the importance of careful risk stratification when considering aggressive immunosuppressive therapy in severe cases.

The clinical course aligns with the preliminary classification criteria for CAPS: rapid progression within one week, multi-organ dysfunction (respiratory, hematologic), and confirmed aPL positivity. Although definitive CAPS requires ≥3 organ systems, the severe and rapidly progressing presentation raises the possibility that early intervention (triple therapy) could have mitigated further progression [3]. In managing CAPS, triple therapy consisting of anticoagulation, high-dose corticosteroids, and plasma exchange plus IVIG (with or without rituximab) has been shown to significantly improve outcomes [3]. In this patient, plasmapheresis or IVIG was withheld due to stability after corticosteroids, though the extracorporeal membrane oxygenation (ECMO) requirement raises the question of whether triple therapy could have prevented deterioration. Future studies should assess early multimodal immunosuppressive therapy in severe APS-associated DAH, especially in probable CAPS.

Persistent aPL positivity places this patient at high thrombotic risk, requiring lifelong anticoagulation [16]. Warfarin (target INR = 2-3) remains the gold standard for high-risk APS patients with prior thrombosis [14]. Prognosis depends on fast recognition and aggressive intervention. Although this condition can be life-threatening, our patient’s successful recovery shows that even severe pulmonary complications may be reversible with timely management. After four years, the patient remains free of APS manifestations. Relapse of CAPS is uncommon. In the only long-term study, 66% of survivors remained asymptomatic on anticoagulation at ~67 months, with no CAPS recurrence, while only 19% of survivors experienced further APS events [17]. Despite favorable outcomes, multidisciplinary collaboration and continuous follow-up remain essential to detect relapse and ensure effective anticoagulation.

## Conclusions

This case highlights a rare and life-threatening manifestation of primary APS presenting as DAH, adding to the scarce literature on this topic. The updated 2023 ACR/EULAR classification criteria underscore the expanding clinical spectrum of APS and reinforce the need for clinicians to maintain a high index of suspicion in patients with unexplained DAH, as timely diagnosis and rapid, high-intensity intervention can significantly impact outcomes.
